# Insertion of the protective APP A673T mutation by CRISPR/Cas9 base editing or PRIME editing.

**DOI:** 10.1093/geroni/igab046.2511

**Published:** 2021-12-17

**Authors:** Jacques Tremblay, Antoine Guyon, Joël Rousseau, Guillaume Tremblay, Francis-Gabriel Begin, Gabriel Lamothe

**Affiliations:** 1 Laval university, Québec, Quebec, Canada; 2 Laval university, Quebec, Quebec, Canada

## Abstract

There is currently no treatment for Alzheimer disease (AD). However, the Icelandic mutation in the APP gene (A673T) has been shown to confer a protection against the onset and development of AD (Jonsson et al. Nature 2012). This single nucleotide mutation in APP exon 16 reduces the cleavage of the APP protein by the beta-secretase by 40% thus preventing the development of AD even in persons more than 95 years old. Our research group has initially shown that the presence of the A673T mutation in an APP gene reduced the secretion of beta-amyloid peptides even if there is also a FAD mutation in the gene. This is the case for 14 different FAD mutations. We have used CRISPR/Cas9 base editing and PRIME editing technologies to insert the A673T mutation in the APP gene. We have compared several different cytidine base editor complexes to achieve the most effective and accurate genome modification possible in HEK293T cells and in SH-SY5Y neuroblastomas. The insertion of the A673T mutation in cells containing the London mutation reduced the secretion of beta-amyloid peptides. We are currently using lentiviral vectors to infect neurons from a mouse model and human neurons induced from fibroblasts of a patient with the London mutation. The insertion of the protective Icelandic mutation in the APP gene using these editing technologies opens a new potential therapeutic avenue not only for Familial Alzheimer’s diseases but also for sporadic Alzheimer’s disease.

